# GPCR signaling via cAMP nanodomains

**DOI:** 10.1042/BCJ20253088

**Published:** 2025-05-13

**Authors:** Rahul Yadav, Manuela Zaccolo

**Affiliations:** 1 Department of Physiology, Anatomy and Genetics, University of Oxford, Oxford, OX1 3PT, United Kingdom

**Keywords:** cAMP signaling, compartmentalization, GPCR, phosphodiesterases, protein kinase A

## Abstract

G protein-coupled receptors (GPCRs) are the largest family of cell surface receptors, mediating essential physiological responses through diverse intracellular signaling pathways. When coupled to Gs or Gi proteins, GPCR modulates the synthesis of 3′-5′-cyclic adenosine monophosphate (cAMP), which governs a wide array of processes, ranging from cellular growth and survival to metabolic regulation. Studies have highlighted that cAMP is not uniformly distributed within cells but instead is compartmentalized into highly localized nanodomains. These nanodomains, mostly regulated by phosphodiesterases (PDEs), play a critical role in enabling signal precision and functional effects that are specific to individual stimuli. GPCRs can initiate distinct cAMP responses based on their localization within the cell, with evidence showing that both receptors resident at the plasma membrane and intracellular receptors—including endosomal, Golgi, and nuclear GPCRs—elicit unique cAMP signaling profiles. This review examines the mechanisms underlying GPCR signaling through cAMP nanodomains. We focus on the role of PDE-mediated cAMP degradation in shaping local cAMP signals, the emerging views on mechanisms that may contribute to signal compartmentalization, and the role of intracellular membrane compartments. By exploring these aspects, we aim to highlight the complexity of GPCR signaling networks and illustrate some of the implications for the regulation of cellular function.

## Introduction

Membrane receptors and their downstream signaling pathways are integral to the regulation of nearly all biological functions in multicellular organisms. Among these, G protein-coupled receptors (GPCRs) represent the largest class. GPCRs are seven transmembrane receptors. The human genome encodes more than 800 GPCRs [1], underscoring their significance in regulating physiological processes. These receptors respond to diverse signals, including sensory inputs, amines, peptides, proteins, lipids, and nucleotides, reflecting their evolutionary adaptability [2]. GPCR transduces the signal input to the intracellular machinery through second messengers, including cAMP and calcium [2], [3]. To initiate signaling, GPCRs interact with heterotrimeric G proteins, intracellular transducers, which consist of three subunits: Gα, Gβ, and Gγ [4]. G proteins act as molecular switches and, upon agonist binding to a GPCR, the receptor facilitates the exchange of GDP to GTP on the Gα subunit, triggering its dissociation from the Gβγ dimer [5], [4], [6]. This separation generates two active signaling entities: the Gα subunit and the Gβγ complex. The 16 Gα subunits are categorized into four main families: Gs, Gi/o, Gq/11, and G12/13. The Gs and Gi families are known for their opposing regulation of adenylyl cyclase (AC), respectively, stimulating and inhibiting the ability of this enzyme to synthesize cAMP (3′-5′-cyclic adenosine monophosphate) from ATP (Adenosine triphosphate) [3], [7], [8]. The Gq family’s primary effector is phospholipase C, which cleaves phosphatidylinositol 4,5-bisphosphate (PIP2) into inositol 1,4,5-trisphosphate (IP3) and diacylglycerol. This reaction leads to calcium release from the endoplasmic reticulum (ER) and the activation of protein kinase C (PKC). Meanwhile, the G12/13 family predominantly signals through the small G protein Rho, which regulates cytoskeletal dynamics and cell motility [7]. cAMP, the first second messenger identified [9], is a small, hydrophilic molecule present across a wide range of living organisms. A large superfamily of phosphodiesterases (PDEs) hydrolyzes the 3′ phosphate bond of cAMP, converting it into 5′-AMP [10]. This hydrolysis by PDEs is the primary mechanism that terminates cAMP signaling [10]. However, efflux of cAMP into the extracellular space through multidrug-resistance protein (gene name ABCC4) transporters has also been described as a process that reduces intracellular cAMP levels [11]. cAMP exerts its effects by initiating a cascade of signaling events that affect the cellular function through interactions with a limited set of effector proteins, including cAMP-dependent protein kinase A (PKA) [12], exchange proteins activated by cAMP [13], cyclic nucleotide-gated ion channels [14], hyperpolarization-activated cyclic nucleotide-gated channels, and Popeye domain-containing proteins [15]. The most extensively studied downstream effector of cAMP is PKA, a highly promiscuous tetrameric enzyme consisting of two regulatory (R) and two catalytic (C) subunits. Upon cAMP binding to the R subunits, the C subunits are activated to phosphorylate a large variety of target proteins, thereby regulating a vast array of diverse cellular functions [16], [17], [18], [19]. Given that a single cell can express up to 100 different GPCRs, many of which signal via cAMP [20], cells face the challenge of accurately decoding multiple inputs from various GPCRs to ensure specificity in downstream signaling, despite relying on a limited number of effectors [21]. A key mechanism that allows cells to solve this problem is spatial segregation through the organization of cAMP nanodomains located within different subcellular compartments. Within these compartments, A-kinase anchoring proteins (AKAPs) organize multiprotein complexes, or signalosomes, which localize PKA in proximity to specific phosphorylation targets. AKAPs can also anchor other signaling components, including PDEs and phosphatases, enabling precise regulation of the amplitude, duration, and functional outcomes of the cAMP signal [22]. Below, we discuss our current understanding of GPCR signaling via cAMP nanodomains while focusing on the established role played by local degradation of cAMP by PDEs and on emerging modalities that may contribute to cAMP compartmentalization.

### GPCR and cAMP

Following GPCR activation, cAMP signaling is primarily initiated at the plasma membrane through the activation of Gα proteins [23]. Notably, numerous receptors can couple to the same Gα protein, and individual receptors can couple to multiple Gα proteins, highlighting the complexity of GPCR signaling [24]. GPCRs are frequently organized into specialized micro- or nanodomains [25], where cytoskeletal scaffold proteins create nanoscopic signaling hotspots that ensure efficient engagement of transducer proteins. These specialized micro- or nanodomains are partially composed of actin fibers, microtubules, and elements of the cytoskeleton, including clathrin-coated pits [26]. Together, these structures form concentrated hotspots on the plasma membrane where receptors and G proteins colocalize. Such organization has been demonstrated with imaging studies using the nanobody Nb37 in CHO[MZ1] (derived from epithelial cells of the Chinese hamster ovary) cells [26]. Nb37 binds to the surface of the alpha-helical (AH) domain of Gαs, where the AH domain is accessible only in the nucleotide-free form of Gαs, representing the catalytic intermediate of G protein activation. In detail, structural studies of Gαs and other Gα subunits have revealed that they consist of two distinct domains: the Ras-like domain (RD)—also known as the GTPase domain—and an AH domain, which is composed of six α-helices embedded within the RD. The RD shares homology with monomeric G-proteins such as p21^ras^ and is a conserved feature across the GTPase superfamily. In contrast, the AH is a unique structural element found exclusively in heterotrimeric G-proteins. Structurally, AH and RD together form a cleft that serves as the binding site for the nucleotide [27]. Nb37 can also be used as a domain marker, allowing tracking of the variable positioning of the small AH region of Gαs [28]. In this study, the α2A-adrenergic receptor (α2A-AR), which strongly couples with the inhibitory G protein (Gi), and a pertussis toxin-insensitive Gαi1 construct were labeled with two distinct organic fluorophores via SNAP5 and CLIP6 tags, respectively. These constructs were transiently expressed in CHO using fast two-color single-molecule microscopy combined with single-particle tracking. The analysis revealed a complex dynamic scenario at the plasma membrane, characterized by high-potential regions (areas where the molecules can easily leave or transition) from which α2A-ARs and Gαi subunits rapidly exited and low-potential regions where they tended to remain trapped. Notably, receptor–G protein interactions occurred preferentially within these shared low-energy areas (regions of the membrane where the energy is minimized, promoting stable and efficient receptor–G protein interactions), or ‘hot spots’ [26]. The organization of GPCR and G protein in hot spots allows direct activation of transducers and subsequent amplification of intracellular signals by precisely co-ordinating a variety of effector proteins and second messengers. For instance, when GPCRs are stimulated at low agonist concentrations, cAMP-rich nanodomains form around the receptors. The spatial confinement of cAMP within these domains is regulated by the cAMP degrading PDEs, as when the PDEs are inhibited, the boundaries between these domains are disrupted [29], [30]. Additionally, scaffolding proteins also contribute to maintaining the spatial precision of GPCR signaling by organizing effector proteins into specialized complexes [25]. This organization facilitates the integration, co-ordination, and processing of cellular signaling events [31]. For example, AKAP5, found to be mostly in association with the plasma membrane, displays the ability to associate with the β_2_-adrenergic receptor [32]. AKAP5 has been shown to interact with PKA holoenzymes containing RIIα or RIIβ, PKC, calmodulin, calcineurin phosphatase (PP2B), AC type V/VI, L-type calcium channels, and β-adrenergic receptors [33]. AKAP5 primarily functions by binding kinases like PKA and phosphatases such as calcineurin to regulate cAMP signaling effectively [34]. Through this, AKAP5 modulates a wide range of cellular processes, including neuronal and glial cell development, differentiation, and migration; the functioning of neural circuits involved in information processing; and the permeability of the blood–brain barrier [35], where disruption of AKAP interactions or dysregulated expression levels has been shown to contribute to the accelerated pathological progression of ischemic stroke [35]. AKAP5 interaction with phosphatase2B (PP2B) and PKA integrates the cAMP oscillations with Ca²^+^ signaling in pancreatic β-cells, thereby regulating glucose-stimulated insulin secretion (GSIS) [36,37]. GSIS involves cell membrane depolarization and the influx of Ca²^+^ through voltage-gated calcium channels [38]. The deletion of AKAP5, or more specifically the disruption of its interaction with PP2B, impairs L-type Ca²^+^ currents and reduces the cytoplasmic accumulation of Ca²^+^ and cAMP in β-cells, inhibiting GSIS [36].

### PDEs and cAMP

The cAMP signal is terminated predominantly via its hydrolysis by PDEs, whose enzymatic activity was initially used to demonstrate the physiological significance of this cyclic nucleotide [39]. The mammalian PDEs are categorized into 11 families, encoded by 21 distinct genes, each exhibiting multiple splice variants with varying subcellular localizations [10,40]. PDE4, PDE7, and PDE8 selectively hydrolyze cAMP, and PDE1, PDE2, PDE3 PDE10, and PDE11 hydrolyze both cAMP and cGMP [10,40], while PDE5, PDE6, and PDE9 selectively hydrolyze cGMP. This specificity has been attributed to a ‘glutamine switch’, a conserved glutamine residue that regulates the binding of the cyclic nucleotide purine ring within the binding domain [41]. The PDE subfamilies are characterized by distinct functional domains, including a relatively conserved catalytic domain and a variety of family and subfamily regulatory domains [40,42]. Primarily, variability between PDE isoforms is found in the N- and C-terminal domains within each subfamily, which are critical for subcellular localization and activity regulation [40,42]. PDEs play a dynamic and sophisticated role in modulating cAMP signaling. This is achieved, in part, through modulation of their activity by post-translational modifications, including phosphorylation and activation by PKA [43,44]. As cAMP levels increase in the cell, PKA can phosphorylate and activate PDEs (for example, PDE3 and PDE4 isoforms), initiating a feedback loop that terminates the cAMP signal [44]. For instance, the localization and trafficking of the PDE10A splicing variant PDE10A2 are regulated by a balance between phosphorylation at threonine-16 (T16) and palmitoylation at cysteine-11 (C11), which respond to local cAMP fluctuations. In PC12 cells (derived from transplantable rat pheochromocytoma) [45], elevation of cAMP activates PKA, leading to PDE10A2 phosphorylation at T16. This phosphorylation disrupts palmitoylation at C11, resulting in accumulation of PDE10A2 in the cytosol, where it degrades cAMP to restore cellular homeostasis. Conversely, with low cAMP levels, PDE10A2 undergoes palmitoylation at C11, promoting its association with intracellular transport vesicles and facilitating its transport to the plasma membrane, where it regulates dopaminergic and glutamatergic signaling pathways [46]. This dynamic regulatory mechanism controls both membrane association and the efficient trafficking of PDE10A2 to distal dendrites [46], showing the critical role of PDEs in spatially regulating cAMP signaling. A detailed discussion on PDEs is beyond the scope of this review. However, several comprehensive reviews are available that thoroughly examine the role of PDEs. For instance, a recent review explores the kinetic properties of PDEs, including K_m_ and V_max_, as well as their involvement in cardiac pathophysiology [47], highlighting the potential of PDEs as therapeutic drug targets.

### cAMP nanodomains

The question of how cells activate stimulus-specific physiological responses despite expressing multiple GPCRs that stimulate similar levels of cAMP has remained unanswered for several decades. The observation that even activation of the same receptor family can elicit different functional effects gave a clear indication of the complexity of the system. A now widely accepted explanation of how hormonal specificity is achieved is the formation of cAMP-dependent ‘signalosomes’, multiprotein complexes that nucleate components of the cAMP signaling pathway to specific subcellular sites and are exposed to spatially restricted pools of cAMP. A prototypical cAMP signalosome may include (i) a cAMP effector, e.g., PKA; (ii) a PDE that degrades cAMP; and (iii) a scaffold protein such as an AKAP, which anchors the complex to a specific subcellular location [48,49]. Under basal conditions, localized PDE activity maintains cAMP levels below the activation threshold for PKA. Upon hormonal stimulation, the elevated cAMP concentration may be sufficient to surpass the rate of PDE-mediated degradation, leading to PKA activation. Subsequently, depending on the specific PDE present, PKA phosphorylation of the PDE may enhance its cAMP hydrolytic activity, restoring cAMP levels to baseline [50]. A good example of how cAMP signalosomes may operate to generate specific cellular effects has been reported for the cAMP response to sympathetic stimulation in the heart. Cardiac myocytes express two isoforms of the β-adrenergic receptor (β-AR), β1-AR and β2-AR. Both are activated by sympathetic hormones (epinephrine/norepinephrine) and initiate Gs-mediated cAMP production but yield distinct functional effects [51,52]. In mouse cardiac myocytes, β1-AR and β2-AR couple with separate cAMP nanodomains, where β1-ARs preferentially engages the PDE4D variant PDE4D9, whereas β2ARs activation recruits the PDE4D5 variant [53]. At resting state, β2AR preferentially associates with PDE4D9. However, upon stimulation, PDE4D9 dissociates from the receptor, while PDE4D5 is recruited to the receptor in a β-arrestin-dependent manner, likely through binding to β2AR/β-arrestin complexes [53]. Under basal conditions, PDE4D9 controls local cAMP concentration and PKA activity near β2-AR, whereas PDE4D5 likely affects β2AR signaling after ligand binding and β-arrestin recruitment. Knockdown of PDE4D9 leads to a substantial increase in the β2-AR-induced cAMP, though the increase remains below that observed with total PDE4 inhibition using rolipram, and results in increased PKA phosphorylation of the receptor under both basal and stimulatory conditions [53]. Overexpression of dominant-negative variant, such as PDE4D-DN, substantially increases the basal contraction rate. These effects of PDE4D9 are thought to be mediated by altered cAMP levels and PKA phosphorylation within β2-AR complexes, while the PDE4D5 effects were suggested to be partially due to the nonspecific increase in cytosolic cAMP and PKA phosphorylation of proteins away from the receptor complexes, as dominant-negative PDE4D5 did not affect PKA phosphorylation of β2-AR [53].

The notion that cAMP has restricted access to distinct intracellular subsets of effectors was proposed over 30 years ago, based on evidence that the two PKA isoenzymes, PKA I and II, localize to different cellular compartments. Specifically, PKA II predominantly associate with the particulate fraction of heart extracts, whereas a larger proportion of PKA I is found in the soluble fraction [54]. These findings led to the hypothesis that epinephrine stimulation selectively activates the holoenzyme in the particulate fraction [51,52]. Although various hormones elevate cAMP in cardiac myocytes, β-adrenergic agonists influence cardiac contractility while prostaglandin does not, supporting the selective activation of a subset of PKA effectors downstream of these two agonists [55,56]. These observations led to the formulation of principles outlining the non-uniform nature of cAMP signaling. Despite being a small, diffusible molecule and therefore expected to equilibrate homogeneously within the cell, it was hypothesized that cAMP does not equally access all PKA isoforms, suggesting the existence of spatially segregated cAMP pools that define distinct signaling compartments [57]. Early experimental evidence consistent with this concept includes the finding that *in vitro* stimulation of local β-ARs located at one extreme of a cardiac myocyte only enhances Ca^2+^ currents in proximity of the activated receptors but not at the opposite extreme of the cell, supporting spatial constraints on cAMP signal propagation [58]. Notably, inhibition of PDEs with IBMX (3-isobutyl-1-methylxanthine) led to a global increase of Ca^2+^ currents across the cell, indicating the key role of cAMP hydrolysis in cAMP compartmentation.

Significant progress in understanding cAMP compartmentalization was made possible by the introduction of molecular tools designed for real-time visualization and monitoring of intracellular cAMP in smooth muscle (BC3Hl) and fibroblast (REF-52) cell lines [59,60]. Unlike previous methods that measured bulk cAMP in cell homogenates at a single time point, these new tools allowed accurate monitoring of the intracellular dynamics of free cAMP with sub-micrometer resolution. Using this approach, direct evidence of cAMP compartmentalization in cardiac myocytes was obtained [61]. This method relies on fluorescence resonance energy transfer (FRET) between a donor and an acceptor fluorophore, each genetically fused to one or two cAMP-binding domains. The sensor design is such that the fluorophores come in close proximity to each other or move further apart upon cAMP binding, altering energy transfer and resulting in a measurable change in emitted fluorescence (ΔFRET). This approach enables detection of signaling events with high temporal and spatial resolution in intact, living cells, preserving the complexity of the intracellular environment [62–66]. The subcellular precision of this technique, combined with its ability to quantitatively track changes in real-time, enabled the first direct demonstration that β-AR activation with norepinephrine does not lead to uniform cAMP distribution within cardiac myocytes. Instead, it forms intracellular gradients, with higher concentrations at specific subcellular sites [61]. In the original study performed in CHO (Chinese hamster ovary) cells, cAMP concentration changes were tracked by monitoring FRET between the PKA RII subunit (linked to a cyan fluorophore) and the PKA C subunit (linked to a yellow fluorophore) [63]. This sensor revealed that the cAMP response induced by norepinephrine was confined to subcellular compartments where PKA is anchored to AKAPs. A variety of cAMP FRET sensors with different designs have been developed since then [62,64,65,67] and multiple studies have confirmed cAMP compartmentalization, both in cardiac myocytes [68–72] and various other cell types. These include for example CFBE (human cystic fibrosis bronchial epithelial cell, derived from a cystic fibrosis patient) [73], mIMCD‐3 (cells exhibiting epithelial morphology, isolated from the kidney of an adult mouse) and HEK293T cells [74], human airway smooth muscle cells (specialized muscle cells found in the airways of the lungs) [75], and pulmonary microvascular endothelial cells (line the small blood vessels (capillaries) in the lungs, forming a crucial barrier for gas exchange) [76]. More recent studies revealed that the size of cAMP compartments can be in the range of nanometers. As their size can be below the resolution limit of standard microscopy (200 nm), targeting the reporters within the nanodomains is necessary to capture the spatial and functional properties of the local cAMP signal [77]. Real-time imaging of cardiac myocytes using targeted reporters demonstrated that distinct subcellular structures, such as the plasmalemma, sarcoplasmic reticulum, and myofilaments—despite being only ∼300 nm apart—experience unique cAMP signaling dynamics in response to catecholamine stimulation [67].

The size of the cAMP pools generated near GPCRs in HEK293 (derived from human embryonic kidney cells) is consistent with the estimated 15–25 nm diameter of a single AKAP:PKA complex [78] and falls within the range where PDEs can effectively regulate cAMP levels [29]. Additionally, the size of these cAMP nanodomains aligns with the dimensions of AKAP:PKA clusters observed at the plasma membrane of CHO cells using super-resolution microscopy, where the average diameter of these clusters is around 100 nm [79]. This suggests that the extension of a cAMP nanodomain is calibrated to be of the size necessary to facilitate the activation of an individual PKA/AKAP signalosomes.

#### Key role of PDEs in cAMP compartmentalization

Physical barriers, such as membranes or organelles, have been proposed as obvious obstacles to cAMP diffusion. While these barriers may be relevant in some circumstances, a large body of evidence supports a critical role of PDEs in shaping intracellular cAMP gradients via differential degradation of cAMP at different locations [48,80–82]. Two primary models have been proposed to explain how PDEs regulate the formation of cAMP nanodomains: the barrier model and the sink model.

**Figure 1:**
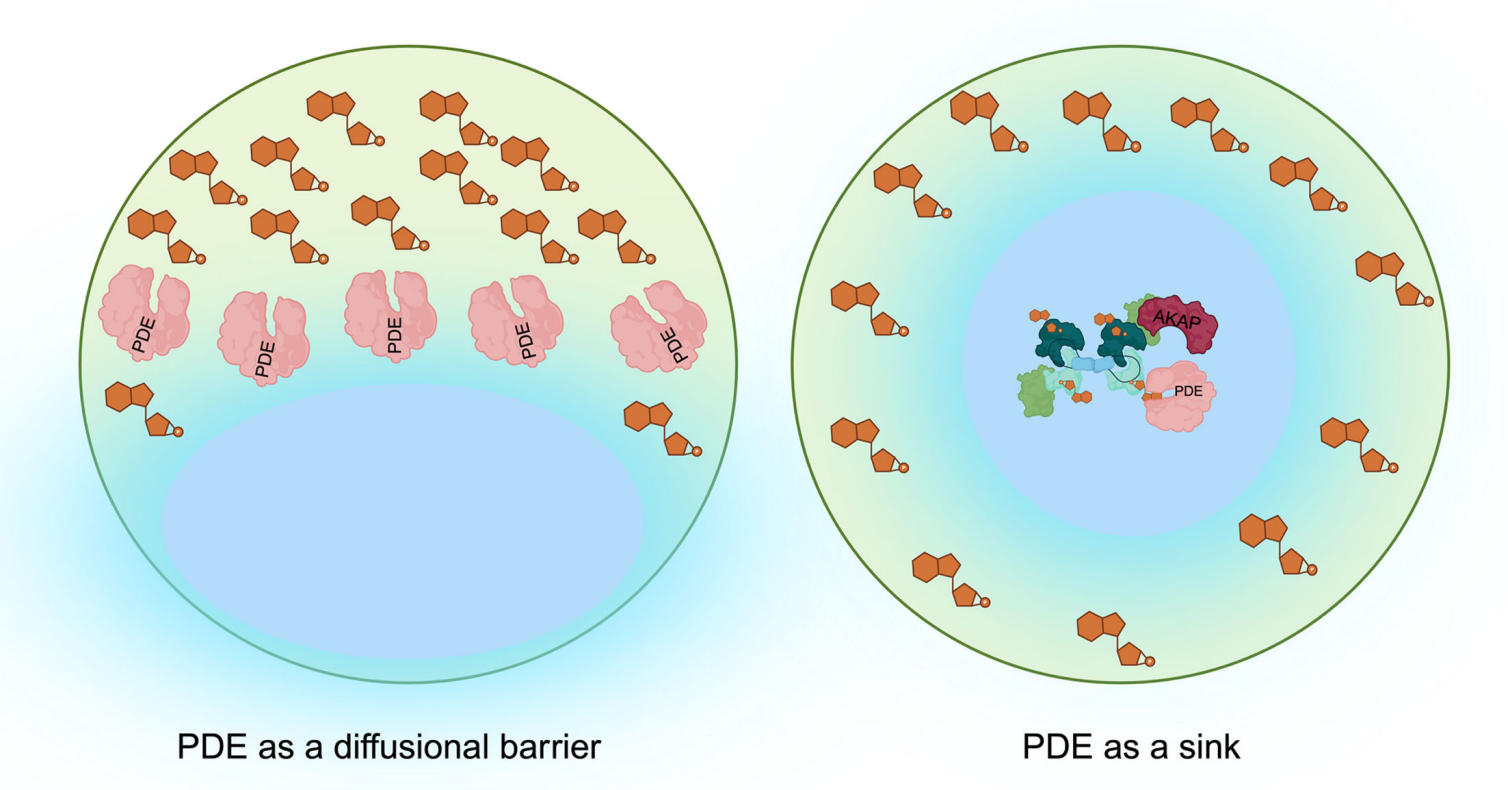
Models for mechanism of action of PDEs. The figures were generated using biorender (Created in BioRender. Zaccolo (2025) https://BioRender.com/c75z717).

The notion that PDEs act as barriers to cyclic nucleotide diffusion stems from initial observations that PDE inhibition results in the broader spreading of the cAMP signal (Figure 1). The early experiments using whole-cell patch recording in frog ventricular myocytes demonstrated that local β-adrenergic stimulation could only be detected at distal L-type Ca^2+^ channels following PDE inhibition with IBMX [58], [83] suggesting the presence of a barrier to cAMP diffusion. Similarly, results showing that stimulation of adenosine receptors can activate the cystic fibrosis transmembrane conductance regulator channel at a distance only in the presence of PDE4 inhibitors [84] were interpreted as consistent with the presence of an enzymatic barrier to cAMP diffusion. Several other studies reported extended diffusion of cAMP signals upon PDE inhibition [85], [61], [86]. In neurons, for example, cAMP signals were shown to be restricted to specific regions near lipid rafts, with functional pools estimated to be between 20 and 200 nm [87]. Immunolocalization studies confirmed PDE4 enrichment in these areas [71], [86], supporting the idea that PDEs function as barriers protecting macromolecular complexes or subdomains from cAMP intrusion. Another example where the notion of a barrier has been invoked involves receptor-associated independent cAMP nanodomains (RAINs). At low agonist concentrations, where receptor occupancy is minimal, GPCRs (such as GLP-1R) create localized cAMP pools near the receptor, extending up to 60 nm away from the membrane. This localized cAMP pool directly contributes to receptor-associated PKA activity, forming a self-sustained and independent signaling unit. The study also suggests that PKA regulatory subunits are required within RAINs for the localized binding and buffering of cAMP. This localization ensures that cAMP remains confined, while also allowing localized PDEs to regulate the size of the GPCR-associated independent cAMP nanodomains [29].

Despite numerous experimental studies [58], [61], [86], [88], [89], [90], [91], [92], [93], [94], along with computational studies [95], [96], indicating that PDEs play a critical role in the compartmentalization of cAMP signaling [72], [97], [98], the model of an enzymatic barrier is not completely satisfactory, as it does not explain how PKA subsets anchored away from the membrane and the receptors that trigger cAMP synthesis could ever become activated. In addition, this model suffers from a major challenge. The core issue lies in the apparent paradox between the rapid diffusivity of cAMP and the relatively slow enzymatic activity of PDEs [99]. The diffusion coefficient of cAMP has been experimentally estimated to be around 40 mm²/s—only about an order of magnitude slower than in water[100] . Given the rapid intracellular diffusion of cAMP, one would expect fast equilibration of cAMP levels throughout the cell, which seems to contradict the concept of cAMP compartmentalization [68], [93], [94]. Considering the reported K_M_ and V_max_ values for PDEs, it is difficult to reconcile the relatively low efficiency of PDE binding and hydrolysis of cAMP with the generation of gradients, even at basal cAMP levels, which are estimated to be around 1 µM in several cell types [99], [101], [85]. This challenge becomes even greater when trying to explain how PDEs could compartmentalize high concentrations of rapidly diffusing cAMP generated in response to hormonal stimulation.

An alternative interpretation to the barrier function is that PDEs may act as ‘sinks’, depleting cAMP in specific locations where they are localized. According to this model, PDEs generate subcellular domains, where cAMP concentrations are kept sufficiently low to prevent PKA activation (Figure 1) [68]. Evidence supporting this notion includes the characterization of a complex comprising the relaxin family peptide receptor 1 (RXFP1), the scaffold protein AKAP79, AC, AC2, β-arrestin 2, and PDE4D3 in HEK293 cells overexpressing RXFP1 [102]. In this model, relaxin induces a biphasic increase in cAMP, with the most sensitive response occurring in the femtomolar range. This response is abolished in the presence of a PDE4 inhibitor [102]. These findings suggest that the PDE4D3–PKA–β-arrestin complex maintains a nanodomain with low cAMP levels, which is crucial for a highly sensitive relaxin response [102]. A similar conclusion has been drawn for β1-adrenergic receptor signaling, where antagonists of β1AR signaling disrupt PDE4 complexes, leading to a localized increase in cAMP near the receptor despite a reduction in bulk cAMP levels [103].

#### Emerging mechanisms for cAMP compartmentalization

cAMP buffering has recently been proposed as a mechanism that contributes to cAMP compartmentalization. cAMP mobility has been measured in the basal state and shown to be minimal [30]. According to these studies, under basal conditions, cells are capable of buffering the majority of their cAMP. Upon minimal activation of ACs, buffering combined with the hydrolyzing activity of PDEs is still sufficient to create a cAMP nanodomain in proximity to the receptor. When PDEs are inhibited and stimuli are more intense, cAMP levels rise sufficiently to surpass the buffering capacity, leading to a progressive increase in cAMP concentrations that eventually affects the entire cell [30].

**Figure 2:**
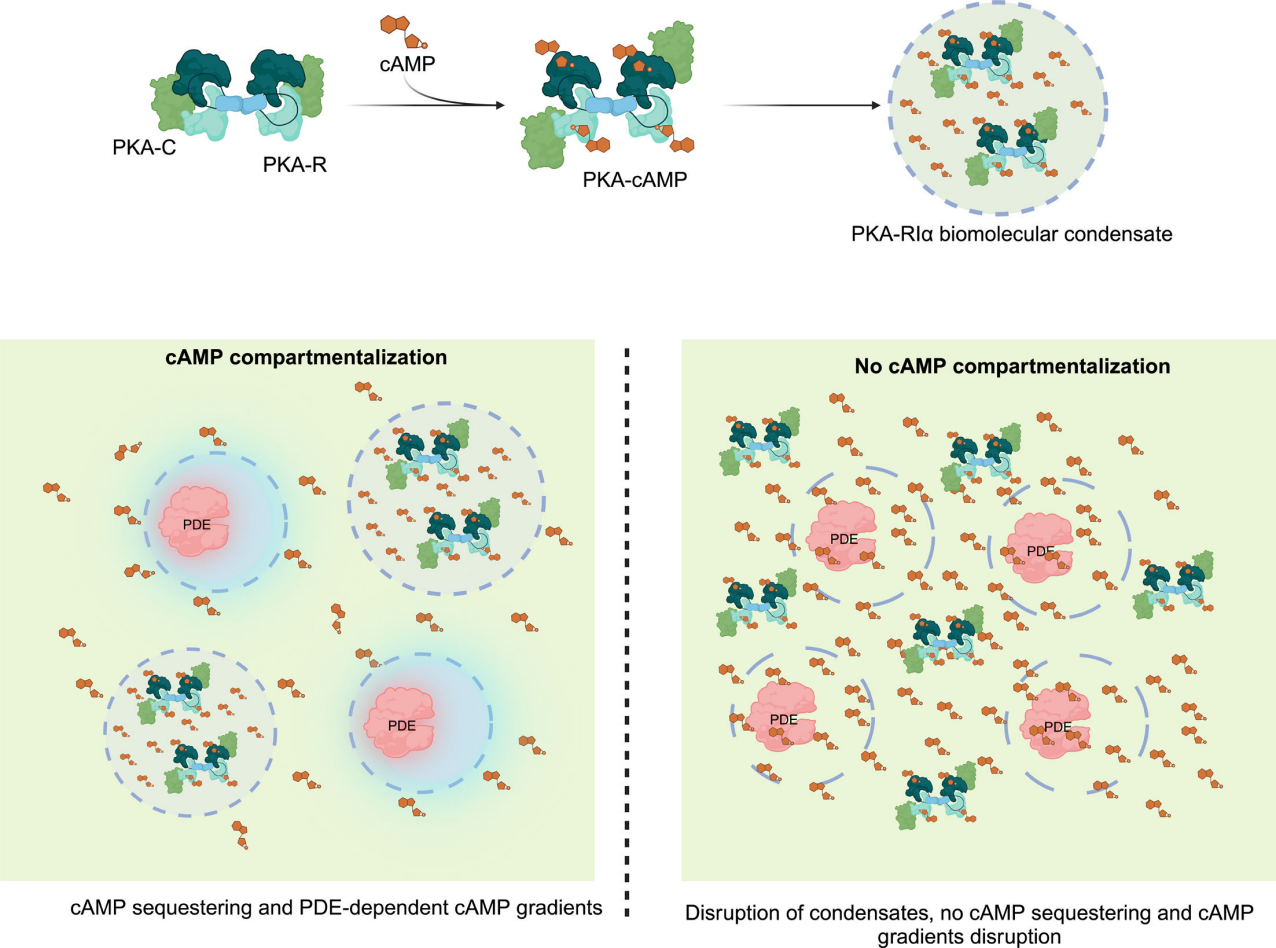
Biomolecular condensation of PKA-PDE-RIα. The figures were generated using biorender (Created in BioRender. Zaccolo (2025) https://BioRender.com/h51c625).

A recent study in HEK293T cells proposed a crucial driver of cAMP compartmentalization is buffering of cAMP through the formation of biomolecular condensates composed of the RI (regulatory) subunit of PKA [104]. The data show that RIα undergoes liquid–liquid phase separation (LLPS) at endogenous levels [105]. These condensates are enhanced in response to cAMP-raising stimuli, forming RIα bodies that contain high levels of cAMP and PKA activity. This dynamic sequestration of cAMP has been proposed to be essential for cAMP compartmentalization and disruption of these condensates and the release of the cAMP they sequester has been suggested to result in loss of PDE-mediated cAMP gradient formation (Figure 2). Interestingly, LLPS of RIα is disrupted by a PKA catalytic fusion oncoprotein present in fibrolamellar carcinoma, resulting in aberrant cAMP signaling and cell transformation, indicating that RIα biomolecular condensates may play an important functional role [106].

##### PDE8-PKA/substrate channeling

**Figure 3:**
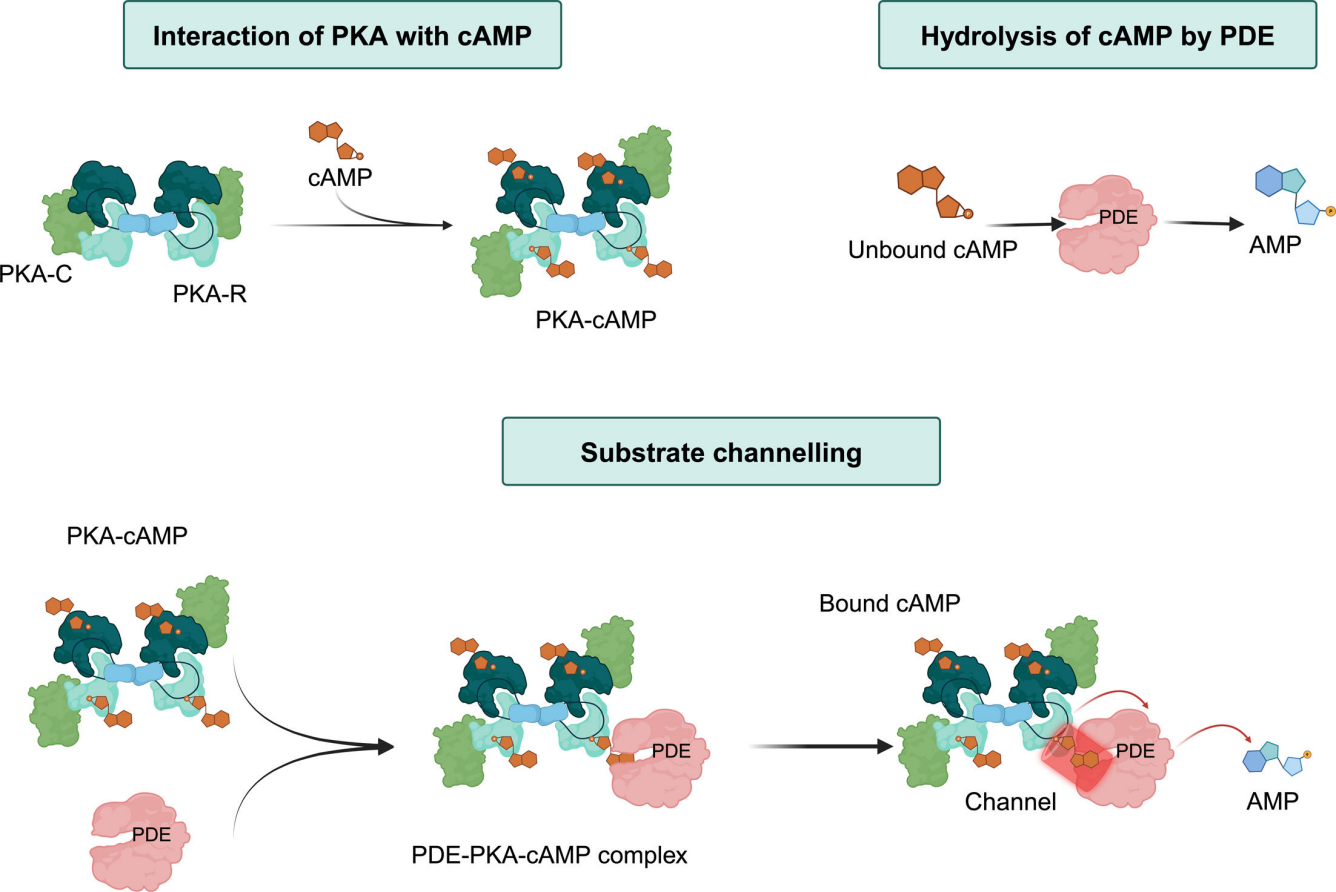
cAMP channeling in a PDE8/PKA-RI complex. The figures were generated using biorender (Created in BioRender. Zaccolo (2025) https://BioRender.com/j24o028).

While PDEs, including PDE8, are generally assumed to hydrolyze unbound, free cAMP, studies suggest that PDEs can hydrolyze cAMP bound to its primary receptor, the R-subunit of PKA, through a molecular channeling mechanism [107], [108], [109]. This model offers an alternative mechanism for the dynamic turnover of cAMP levels. The concept of substrate channeling, commonly associated with metabolic enzymes, refers to supramolecular complexes that restrict diffusion of reaction intermediates by forming channels between associated proteins [110]. Channeling allows enzyme reactions to occur in a co-ordinated manner without the release of ligands or intermediates into the surrounding solvent (Figure 3). In the context of cAMP signaling, channeling may support the rapid and precise hydrolysis of cAMP bound to PKA by forming a PDE/PKA-R complex [108], [111]. This mechanism has been described in some detail *in vitro* for PDE8 and seems to involve high-affinity binding of cAMP to the R-subunit. The molecular channel would enable PDE8 to have direct access to cAMP, when tightly bound to PKA-R subunits and facilitate the translocation of cAMP to the enzyme active site for hydrolysis. This mechanism would contribute to the robustness of the cAMP signaling response, ensuring that the cAMP-PKA pathway is activated only during significant cAMP fluxes, such as those observed during hormonal stimulation, allowing for adaptation to steady-state cAMP levels [112].

### GPCR in intracellular membrane compartments and their role in cAMP signaling

Traditionally, GPCRs have been thought to function exclusively at the cell surface, transmitting signals in response to extracellular stimuli. However, recent studies indicate that GPCRs are also capable of signaling from intracellular membrane compartments, including endosomes, the Golgi apparatus, and nuclear membranes [24], [113], [114]. Notably, intracellular GPCR signaling often leads to distinct functional outcomes compared with plasma membrane signaling, despite often utilizing the same G protein effectors and second messengers, supporting a role of signaling from internal membranes in determining specificity of response. Within this paradigm, the generation of cAMP from intracellular GPCRs would provide a local source of second messenger that could feed and activate selectively signalosomes localized nearby.

#### Signaling from endosomal GPCRs

Following activation and internalization from the plasma membrane, many GPCRs enter the endosomal pathway and are sorted within endosomal compartments through a highly regulated process, determining whether they can either recycle back to the plasma membrane or be directed towards lysosomal degradation [115,116]. The sorting process can involve receptor-specific amino acid motifs, post-translational modifications, such as phosphorylation and ubiquitination, and interactions with endosome-associated proteins [117]. The internalization is primarily mediated by clathrin-coated vesicles, facilitated by the interaction of β-arrestin-1 or 2 with the clathrin adaptor protein AP-2 [118,119]. Internalization of GPCRs from the plasma membrane to endosomes was traditionally viewed as a mechanism for signal termination and receptor desensitization [120,121]. However, the discovery that GPCR-mediated G protein signaling can persist from endosomes [122] has revealed a more nuanced role for receptor internalization. One notable example involves the luteinizing hormone receptor (LHR), where sustained cAMP signaling by receptor internalization is essential for the meiotic progression of oocytes within ovarian follicles. LHR expression is predominantly restricted to the two outermost cell layers of the mature ovarian follicle, the theca and mural granulosa cells. Luteinizing hormone (LH) signaling occurs through a GPCR selectively expressed in these external follicular layers, which enables the receptor to trigger distinct temporal and spatial phases of cAMP signaling[123] . The LHR stimulation induces two distinct phases of cAMP signaling in the cells surrounding the oocyte: first a reversible cAMP signal is generated, followed by a persistent increase in cAMP levels after internalization of LHR has occurred. The disruption of LHR internalization abolishes the second, persistent phase of cAMP signaling, which partially inhibits the resumption of oocyte meiosis [123], suggesting that sustained cAMP signaling from internalized LHRs is critical for transmitting LH signals to follicle cells and the oocyte.

#### Signaling from GPCR localized to the Golgi apparatus

GPCRs can also undergo retrograde transport to the Golgi apparatus or may be retained within the Golgi during biosynthetic trafficking before being delivered to the plasma membrane [124]. Retrograde trafficking from endosomes to the trans-Golgi network (TGN) for the recycling and retrieval of protein cargos/transmembrane proteins is frequently mediated by the retromer complex [124]. The retromer is a highly conserved, cytosolic/peripheral membrane hetero-pentameric protein complex present in all mammalian cells. It consists of two endosomal membrane-bound sorting nexins (Snx1 and Snx2), proteins that contribute to membrane recruitment and formation of recycling tubules [125], and a soluble heterotrimer (Vps26, Vps29, and Vps35) that recognizes cargos [126–129]. GPCRs, such as the parathyroid hormone receptor and thyroid-stimulating hormone receptor (TSHR), have been shown to traffic retrogradely to the TGN [130], [131]. While not all GPCRs are recycled through the TGN, all GPCRs are integral membrane proteins and must transit through the Golgi apparatus, where they undergo crucial post-translational modifications, such as glycosylation, prior to their insertion into the plasma membrane [132]. Although many GPCRs pass transiently through the Golgi, certain receptors, like the delta-opioid receptor (DOR) and the β1-adrenergic receptor, display steady-state localization within the Golgi [133], [134]. The export of GPCRs from the Golgi is tightly regulated by various factors, including receptor-specific amino acid motifs, interactions with trafficking proteins, and lipid compositions of the Golgi membrane [135], [136], [137], [138]. The functional significance of Golgi-localized GPCR signaling is evident from the distinct cellular effects it elicits compared with signals generated by GPCRs located at the plasma membrane. One striking example comes from the studies on TSHR, which demonstrate that cAMP and PKA signaling can persist in the Golgi up to 10 minutes post-receptor internalization, and this sustained signaling can be abrogated by treatment with inhibitors of endocytosis [131]. In thyroid cells, internalized TSHR retrogradely traffics to the TGN and activates endogenous Gs proteins within retromer-coated compartments. This process is crucial for inducing a late-phase cAMP/PKA response localized at the Golgi/TGN [131]. Importantly, this localized signaling at the Golgi is required for efficient activation of downstream targets such as cAMP response element-binding protein (CREB), a key transcription factor. Disruption of this TSH-induced phosphorylation pathway—whether by blocking receptor internalization, inhibiting PKA II or its localization to the Golgi/TGN, silencing retromer, or disrupting Golgi/TGN organization—significantly impairs CREB phosphorylation [131]. These processes highlight that retrograde trafficking to the TGN is not simply a means of receptor recycling but instead a critical process that positions GPCRs near the nucleus to activate local Gs protein-mediated cAMP/PKA signaling.

#### Signaling from GPCRs localized to the nuclear membrane and endoplasmic reticulum

GPCRs can localize to nuclear membranes [139] and associated ER membranes either following synthesis or after internalization from the plasma membrane [140]. The localization to the nuclear membranes can be mediated by classical nuclear localization sequences (NLS) and/or by interactions with importin proteins [140]. Nuclear targeting of GPCRs via these mechanisms has been observed following internalization, as demonstrated for the protease-activated receptor 2 and the oxytocin receptor [140]. Alternatively, GPCRs, such as the α1-adrenergic receptors and platelet-activating factor receptor may be targeted directly to the nucleus after synthesis [140], [141], [142], [143]. The protease-activated receptor 4 uses classical RXR (arginine-X-arginine, where X can be any amino acid) motifs for retention in the ER. These motifs are recognized by the COP-I (coatomer protein I) protein complex, which plays a role in the retention of GPCRs within the biosynthetic pathway [144], [145]. Interestingly, similar COPI-interacting motifs are also found in GPCRs localized to the Golgi apparatus, such as the DOR [137], [146]. However, the metabotropic glutamate receptor 5 (mGluR5) achieves nuclear localization through non-NLS sequences, a non-canonical mechanism to reach the nucleus, potentially interacting with chromatin to retain its nuclear presence [147]. Signaling from nuclear membrane-localized GPCRs, such as mGluR5 in neurons, provides a compelling example of how intracellular signaling can diverge [148]. For example, stimulation of either cell surface or intracellular mGluR5 triggers the phosphorylation typical of plasma membrane-based signaling. In striatal neurons, both plasma membrane-localized and intracellular mGluR5 signaling are capable of activating the transcription factor CREB [148]. The activation of mGluR5 at these two locations generates different Ca²^+^ signaling patterns, leading to unique cellular responses of c-Jun N-terminal kinase, Ca²^+^/calmodulin-dependent protein kinase, and CREB. However, only intracellular mGluR5 activation results in the phosphorylation of extracellular signal-regulated kinases 1 and 2 and Elk-1, a transcriptional regulator [148]. In striatal neurons, intracellular mGluR5 is both present and functional in hippocampal neurons, where it can cause dendritic Ca^2+^ rises that differ in amplitude from cell surface receptor activation. It has also been shown that intracellular mGluR5 can mediate protein-synthesis-dependent long-term depression (LTD), whereas cell surface mGluR5 is involved in both LTD and Long-term potentiation [148], highlighting the diverse physiological effects that can arise from GPCR signaling at intracellular membranes.

## Future perspectives

The discovery of novel mechanisms governing cAMP compartmentalization opens several exciting avenues for future research. One critical area of focus is the further exploration of LLPS in the regulation of cAMP nanodomains. The involvement of biomolecular condensates in cellular signaling pathways is a relatively new concept, and future studies will need to investigate how LLPS integrates with other mechanisms, such as PDE activity or PKA-RI subunits interactions with AKAPs, to shape cAMP dynamics. Additionally, understanding the conditions that promote or disrupt LLPS in disease contexts could reveal new therapeutic avenues.

Another promising direction is the study of substrate channeling between PDEs and PKA. While this concept is well-established in metabolic pathways, its application to cAMP signaling is still in its infancy.

Finally, the role of cAMP buffering in regulating cAMP diffusion warrants further exploration. While recent studies have demonstrated its importance, the full extent of its impact on cAMP compartmentalization remains unclear. Future research could focus on identifying additional cAMP-binding proteins involved in buffering and how these interactions change under various physiological and pathological conditions.

As our understanding of cAMP nanodomains continues to evolve, integrating these novel mechanisms with a deeper knowledge of GPCR signaling from different subcellular locations will be crucial for developing a comprehensive model of GPCR signaling via cAMP compartmentalization.

## Abbreviations

AC: adenylyl cyclase
AH: alpha-helical
AR: adrenergic receptor
C11: cysteine-11
CREB: cAMP response element-binding protein
DOR: delta-opioid receptor
ER: endoplasmic reticulum
FRET: Fluorescence Resonance Energy Transfer
GPCR: G protein-coupled receptors
GSIS: glucose-stimulated insulin secretion
LH: Luteinizing hormone
LHR: luteinizing hormone receptor
LLPS: liquid–liquid phase separation
LTD: long-term depression
NLS: nuclear localization sequences
PDE: phosphodiesterases
PKA: protein kinase A
PKC: protein kinase C
PP2B: phosphatase2B
RAINs: receptor-associated independent cAMP nanodomains
RD: Ras-like domain
RXFP1: relaxin family peptide receptor 1
T16: threonine-16
TGN: trans-Golgi network
TSHR: thyroid-stimulating hormone receptor
mGluR5: metabotropic glutamate receptor 5
α2A-AR: α2A-adrenergic receptor
β-AR: β-adrenergic receptor
